# Evidence for a Causal Association Between Human Cytomegalovirus Infection and Chronic Back Pain: A One‐Sample Mendelian Randomization Study

**DOI:** 10.1002/jsp2.70063

**Published:** 2025-04-08

**Authors:** Maryam Kazemi Naeini, Maxim B. Freidin, Isabelle Granville Smith, Stephen Ward, Frances M. K. Williams

**Affiliations:** ^1^ Department of Twin Research and Genetic Epidemiology, School of Life Course and Population Sciences King's College London London UK; ^2^ Pain Management Centre Guy's and St Thomas' NHS Foundation Trust London UK

**Keywords:** causal association, chronic Back pain, cytomegalovirus, Epstein–Barr virus

## Abstract

**Background:**

Chronic back pain (CBP) is a major cause of disability globally. While its etiology is multifactorial, specific contributing genetic and environmental factors remain to be discovered. Paraspinal muscle fat has been shown in human and preclinical studies to be related to CBP. One potential risk factor is infection by cytomegalovirus (CMV) because CMV is trophic for fat. CMV may reside in the paraspinal muscle adipose tissue. We set out to test the hypothesis that previous CMV infection is linked to CPB using a one‐sample Mendelian randomization (MR).

**Method:**

The sample comprised 5140 UK Biobank participants with information about CMV serology and CBP status. A one‐sample MR based on independent genetic variants predicting CMV positivity was conducted in Northern European participants. To validate the association further, the MR study was repeated using a CMV polygenic risk score (PRS). As a negative control for confounding and spurious causal inference, we used Epstein–Barr virus (EBV) serology, because EBV is another common viral infection but is not trophic for adipose tissue.

**Results:**

A genome‐wide association study for CMV seropositivity revealed 86 independent SNPs having p‐value < $2\times 10^{-4}$ that have been used to define genetically‐predicted categories of CMV infection risk. The CMV predicted categories were found statistically significantly associated with CBP (OR = 1.150; 95% CI: 1.005–1.317, *p*‐value = 0.043). Stronger significant results were obtained using the PRS for CMV seropositivity (OR = 1.290; 95% CI: 1.133–1.469, *p*‐value = 12E‐4). No such association was seen between EBV and CBP.

**Conclusion:**

Our results provide evidence for a causal relationship between CMV infection and CBP. Further investigation is warranted to get insight into the mechanism by which CMV might contribute to the pathogenesis of CBP.

## Graphical abstract

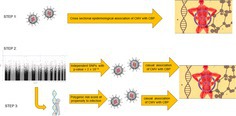

## 1 Introduction

Chronic back pain (CBP), a common musculoskeletal problem that affects people of all ages, has become a leading cause of disability recently. Years of disability due to low back pain have increased by more than 50% for the last 30 years, particularly in countries with low or middle incomes [1].

Epstein–Barr virus (EBV) and cytomegalovirus (CMV) are very common human herpesviruses, and initial infection usually takes place in childhood and is often asymptomatic. The pathogenesis of these infections is complex, CMV causes direct tissue damage, and EBV involves an active immune system [2]. Globally, CMV and EBV infections affect over 50% and over 90% of the population, respectively [3, 4]. There is some evidence of an association between EBV and chronic fatigue syndrome, myalgic encephalomyelitis/chronic fatigue syndrome, and pain intensity after an acute EBV infection [5, 6]. Some studies showed there is a relation between paraspinal muscles and low back pain and spine disc degeneration [7, 8]. Adipose tissue in and around the spine‐supporting muscles, notably the paraspinal muscles, may contribute to CBP. At the same time, the human adipose tissue enhances active CMV infection [9]. CMV may target adipocytes, so we hypothesized that adipose tissue CMV infection may play a role in the etiology of CBP. To the best of our knowledge, no studies focused on CMV infection influencing CBP. The aim of this study was to investigate the epidemiological and potential causal association between previous CMV infection and CBP while considering EBV exposure as a control.

## 2 Materials and Methods

### 2.1 Participants and Measurements

UK Biobank was used for the sample. Using baseline blood samples from 9724 randomly selected volunteers, a multiplex serology panel assessing immunoglobulin G (IgG) antibody response to antigens from 20 infectious pathogens, including CMV and EBV, was conducted in 2016. We considered as participants all volunteers who had results from these assays and CBP status available. Individuals were classified as seropositive if antibody responses to two of the three CMV antigens reached predetermined seroreactivity median fluorescence intensity thresholds, based on antigens (pp28, pp52, and pp150 N‐terminus). EBV seropositivity for EBV was defined as positive if two or more of antigen VCA p18 > 250, antigen EBNA‐1 > 250, antigen ZEBRA > 100, and antigen EA‐D > 100.

EBV measured concurrently with CMV in the UK Biobank—was employed as a negative control. This approach allowed us to assess potential confounding by other viral exposures and to ensure the robustness of the observed association between CMV seropositivity and chronic back pain.

Initial assessment of CBP in UKBB was conducted during 2006–2010 in 141 976 participants. Chronic back pain phenotype was defined using field ID 6159, with this question: “Pain type(s) experienced in last month” and the following question: “Have you had back pain for more than 3 months?” Participants who mentioned they experienced back pain that lasted more than 3 months were considered as cases of CBP. All other participants were considered as controls.

### 2.2 Data Analysis

Information on CMV, EBV seropositivity, and CBP status was available for 5140 participants. Logistic regression modeling was used to investigate the epidemiological association between CMV and EBV seropositivity with CBP while adjusting for age, sex, BMI, ethnicity, and socio‐economic status.

To reveal genetic variants for Mendelian randomization (MR), we carried out a genome‐wide association study (GWS) for CMV and EBV seropositivity using the assayed sample of 8808 Northern Europeans having CMV and EBV seropositivity results. The GWAS was performed with imputed variants using GCTA, adjusted for sex and age. The criteria to filter variants were MAF > 0.005, imputation INFO score > 0.7, and missingness rate < 0.02.

However, the limited sample size precluded the identification of a sufficient number of independent SNPs at the conventional genome‐wide significance threshold.

Therefore, we used a more liberal p‐value threshold of *p* < 5 × 10^−4^ to capture additional potentially informative variants. Although this threshold may include variants with weaker evidence, we mitigated this concern by conducting a one‐sample MR analysis using individual‐level data, which enhances statistical power under these constraints.

To identify important independent genetic variants linked to our traits, we performed linkage disequilibrium (LD) clumping using R's ieugwasr tool. To ensure that variations within this window were assessed for LD, the clumping method was carried out using a distance threshold of 10 000 kb. Variants were deemed to be in high LD if their pairwise r2 value was more than 0.01. To ascertain LD structure, we designated a reference population, such as European ancestry from the 1000 Genomes Project.

In a one‐sample MR study, we used instrumental variable analysis with two‐stage least‐squares regression to investigate the causal relationship between genetically determined CMV and EBV using the ivtools package in R.

Furthermore, to validate our MR findings, we constructed polygenic risk scores (PRS) based on the selected SNPs. The PRS provided a composite measure of genetic predisposition to CMV seropositivity and was used to replicate our primary MR results. We used PRSice2 [10] for the calculation of PRS for all the individuals based on GWAS *p*‐value threshold 5 × 10^−6^. Therefore, the CMV PRS for each individual was used in a two‐stage least‐squares regression, first for predicting CMV categories determined by PRS and then using the estimated CMV categories as a predictor of CBP. The GWAS summary statistics will be made publicly available at Zenodo at the time of publication.

## 3 Results

Of the 5140 participants included in our study, 54.6% were female and 45.4% were male. The median age of participants was 58 years (IRQ: 50–63), and the BMI median was 26.82 kg/m2 (IRQ: 24.27–30.02), and 95.2% of them were of Northern European ancestry (Table 1). There was a statistically significant association between CMV (*p*‐value = 0.001) and EBV (*p*‐value = 0.046) with CBP after adjusting for age, sex, BMI, ethnicity, and socio‐economic status (Table 2). GWAS of CMV outcome using 8883 Northern European samples followed by clumping revealed 86 SNPs with a *p*‐value < 5 × 10^−4^ for the one‐sample MR. SNPs were used to elaborate on genetically predicted categories of risk for CMV infection. The predicted categories were shown to be significantly associated with CBP. A causal association was confirmed by including PRS for CMV as a predictor of CBP in the MR analysis (Table 2).

**TABLE 1:** The basic characteristics (*n* = 5140).

Variables	Descriptive statistics
Sex (female)	2805 (54.6%)
Age (years)	58 (IRQ: 50–63)
BMI (kg/m2)	26.82 (IRQ: 24.27–30.02)
Ethnicity (Northern European)	4891 (95.2%)
Townsend deprivation index	−2.17 (IRQ: −3.72–0.51)
CBP (yes)	1413 (27.5%)
CMV (yes)	2961 (57.6%)
EBV (yes)	4846 (94.3%)

*Note:* Legend to Table 1: IQR: interquartile range, BMI: body mass index.

**TABLE 2:** Association analysis between CMV and CBP.

Association	OR	CI	*p*
Epidemiological association	1.30	1.14–1.47	0.001
Causal association based on 86 independent SNPs	1.15	1.00–1.31	0.043
Causal association based on PRS	1.29	1.13–1.46	12E‐4

*Note:* Legend to Table 2: OR: odds ratio, CI: confidence interval.

GWAS of EBV using 8800 Northern European samples followed by clumping revealed 50 SNPs with *p*‐value < 5 × 10^−4^ for the one‐sample MR. Genetically determined probabilities of risk for EBV infection were elaborated using the SNPs. There was no significant relationship between the predicted probabilities of EBV infection and CBP (*p*‐value = 0.169).

## 4 Discussion

The results of the one‐sample MR analysis using both individual genetic variants and PRS provide evidence for a positive causal relationship between CMV infection and CBP in a Northern European population. However, while the logistic regression result indicated a significant association between EBV, no causal association was found using MR analysis for EBV as a negative control in our investigation; the observed causal association between CMV and CBP is most likely genuine.

There are several hypothetical ways by which CMV may cause CBP. One of them is the impact of CMV on intervertebral disc degeneration (IVDD) which is the major risk factor for CBP, with individuals with the highest IVDD having a threefold greater risk. Alpantaki et al. detected high CMV infection incidence in intervertebral disc and identified herpes virus DNA in the intervertebral disc of individuals with lumbar disc herniation, suggesting that herpes viruses may play a role in the etiology of degenerative disc disease [11]. Another link between CMV and CBP includes t adipose tissue in and around the muscles that support the spine and are known to contribute to CBP. In CBP, the so‐called paraspinal muscles exhibit increased adipose deposition and atrophy [12]. Unilateral back pain is associated with paraspinal fat infiltration on the same side as the symptoms, and animal studies support the concept that pain is produced by muscle fat infiltration [13, 14]. Human adipose tissue stimulates active CMV infection, and CMV may target adipocytes. Adipose tissue infection may play an important role in the etiology of CMV condition [15]. Contreras et al. showed that human white adipose tissues as a large reservoir of CMV‐specific T lymphocytes, most likely used to manage latent viral reactivation [9].

While our findings provide novel evidence for a potential causal link between CMV infection and CBP, further research is needed to confirm and expand upon these results. Given the limitations in available datasets with CMV and EBV serology data, future studies should focus on investigating this association in larger and more diverse populations to enhance statistical power and generalizability.

Additionally, exploring the biological mechanisms underlying this relationship is crucial. One promising avenue is CMV's known tropism for adipose tissue, particularly in the paraspinal muscles. Investigating whether CMV infection directly contributes to muscle fat infiltration or other structural changes associated with CBP could provide valuable mechanistic insights. Longitudinal studies incorporating imaging data, inflammatory markers, and immune profiling would help further elucidate the role of CMV in CBP pathogenesis.

Future research should aim to refine genetic instruments for CMV infection by leveraging larger GWAS datasets as they become available. Improved genetic proxies will strengthen causal inference and allow for more robust Mendelian randomization analyses. These efforts will not only validate our findings but also inform potential preventive and therapeutic strategies targeting CMV‐associated pathways in CBP.

We also acknowledge the importance of accounting for potential confounders, including differences in immune status and comorbid conditions, which may influence both CMV infection risk and CBP prevalence. While our study controlled for key demographic and lifestyle factors known to impact these associations, direct measures of immune function were not available. The UK Biobank includes serology data for several viral infections, such as herpes simplex virus type 1 (69.8%), herpes simplex virus type 2 (16.2%), varicella‐zoster virus (92.5%), EBV (94.7%), and others, but the prevalence of some infections, such as human immunodeficiency virus (0.2%) and human t‐lymphotropic virus type 1 (1.6%), is low, making them less significant for controlling immune status in our analysis. Given these sample size limitations, we used EBV as a negative control, as it is more commonly observed and allows for a more robust comparison.

Future studies should aim to incorporate additional immune markers, such as white blood cell counts, cytokine levels, and C‐reactive protein, to further evaluate immune function as a potential confounder in the relationship between CMV and CBP.

The primary limitation of our study, as we mentioned, is the small sample size employed for the GWAS due to the lack of studies evaluating CMV and EBV traits. Also, due to this limitation, we did not have any other studies to utilize as target populations to calibrate PRS, which may have resulted in overfitting in the first regression stage of MR using PRS.

## 5 Conclusion

Our study showed an increased risk of CBP in those with CMV infection. Given the small scale of the study as well as the use of the one‐sample MR instead of a more robust two‐sample MR warrants further investigation both from epidemiological and mechanistic perspectives. If confirmed in further studies, these results may be useful to develop new diagnostic and treatment approaches for CBP considering CMV infection.

## Author Contributions

Maryam Kazemi Naeini contributed to study design, methodology, data analysis, manuscript drafting. Maxim B. Freidin contributed to methodology, data analysis, manuscript drafting. Isabelle Granville Smith data curation, manuscript drafting. Stephen Ward contributed to study design, manuscript drafting. Frances MK Williams contributed to study design, methodology, manuscript drafting, and supervision.

## Conflicts of Interest

The authors declare no conflicts of interest.
